# Supplementary material for: Modeling glioblastoma heterogeneity as a dynamic network of cell states
Source: Mol Syst Biol. 2021 Sep 16;17(9):e10105. doi: 10.15252/msb.202010105 (PMC8444284; doi:10.15252/msb.202010105)
Supplement: Supplementary file 6 — Source Data for Figure 5 [file MSB-17-e10105-s004.zip › Figure5A_sourcedata/GSEA_3017/nosigdb_stateD.GseaPreranked.1622795010397/NB_KI_CLUSTER6.html]

Details for gene set NB\_KI\_CLUSTER6[GSEA]

|  || Dataset | state23017 |
| Phenotype | NoPhenotypeAvailable |
| Upregulated in class | na\_pos |
| GeneSet | NB\_KI\_CLUSTER6 |
| Enrichment Score (ES) | 0.13822922 |
| Normalized Enrichment Score (NES) | 1.2973503 |
| Nominal p-value | 0.13636364 |
| FDR q-value | 0.3193172 |
| FWER p-Value | 0.741 |
Table: GSEA Results Summary

  

Fig 1: Enrichment plot: NB\_KI\_CLUSTER6      
 Profile of the Running ES Score & Positions of GeneSet Members on the Rank Ordered List

  

| PROBE | GENE SYMBOL | GENE\_TITLE | RANK IN GENE LIST | RANK METRIC SCORE | RUNNING ES | CORE ENRICHMENT || 1 | AC027031.2 |  |  | 13 | 0.494 | 0.0167 | Yes |
| 2 | SQLE |  |  | 25 | 0.421 | 0.0310 | Yes |
| 3 | AL118516.1 |  |  | 42 | 0.368 | 0.0384 | Yes |
| 4 | GDF11 |  |  | 97 | 0.294 | 0.0118 | Yes |
| 5 | CHD7 |  |  | 116 | 0.278 | 0.0127 | Yes |
| 6 | HNRNPLL |  |  | 177 | -0.252 | -0.0209 | Yes |
| 7 | MAN1A2 |  |  | 196 | -0.253 | -0.0214 | Yes |
| 8 | PMF1 |  |  | 222 | -0.256 | -0.0272 | Yes |
| 9 | CNTLN |  |  | 225 | -0.256 | -0.0148 | Yes |
| 10 | FAM172A |  |  | 250 | -0.260 | -0.0196 | Yes |
| 11 | CEP44 |  |  | 260 | -0.260 | -0.0125 | Yes |
| 12 | PIK3R3 |  |  | 277 | -0.261 | -0.0109 | Yes |
| 13 | SNX13 |  |  | 301 | -0.264 | -0.0147 | Yes |
| 14 | ZC3H14 |  |  | 304 | -0.264 | -0.0018 | Yes |
| 15 | P4HTM |  |  | 313 | -0.265 | 0.0063 | Yes |
| 16 | DOCK7 |  |  | 321 | -0.266 | 0.0153 | Yes |
| 17 | ARMC1 |  |  | 326 | -0.266 | 0.0266 | Yes |
| 18 | CYFIP2 |  |  | 334 | -0.267 | 0.0357 | Yes |
| 19 | HGSNAT |  |  | 342 | -0.267 | 0.0447 | Yes |
| 20 | AKT3 |  |  | 378 | -0.271 | 0.0318 | Yes |
| 21 | ATXN2 |  |  | 389 | -0.272 | 0.0388 | Yes |
| 22 | CSGALNACT1 |  |  | 391 | -0.272 | 0.0529 | Yes |
| 23 | NAGK |  |  | 400 | -0.273 | 0.0614 | Yes |
| 24 | RIF1 |  |  | 403 | -0.274 | 0.0748 | Yes |
| 25 | NUP107 |  |  | 422 | -0.276 | 0.0756 | Yes |
| 26 | BNIP3L |  |  | 431 | -0.277 | 0.0844 | Yes |
| 27 | CEP97 |  |  | 474 | -0.282 | 0.0666 | Yes |
| 28 | FBXW7 |  |  | 494 | -0.284 | 0.0671 | Yes |
| 29 | ANKRD36C |  |  | 501 | -0.285 | 0.0779 | Yes |
| 30 | MTUS1 |  |  | 508 | -0.286 | 0.0888 | Yes |
| 31 | TSPAN5 |  |  | 513 | -0.287 | 0.1013 | Yes |
| 32 | HDAC9 |  |  | 548 | -0.291 | 0.0903 | Yes |
| 33 | MLF1 |  |  | 585 | -0.297 | 0.0780 | Yes |
| 34 | HERC2 |  |  | 591 | -0.297 | 0.0903 | Yes |
| 35 | ITFG1 |  |  | 597 | -0.299 | 0.1026 | Yes |
| 36 | GTF2A1 |  |  | 627 | -0.306 | 0.0964 | Yes |
| 37 | TERF1 |  |  | 684 | -0.316 | 0.0694 | Yes |
| 38 | CCNL2 |  |  | 702 | -0.320 | 0.0734 | Yes |
| 39 | TCEAL3 |  |  | 711 | -0.321 | 0.0846 | Yes |
| 40 | PTAR1 |  |  | 730 | -0.325 | 0.0881 | Yes |
| 41 | VPS41 |  |  | 736 | -0.327 | 0.1020 | Yes |
| 42 | FER |  |  | 740 | -0.329 | 0.1176 | Yes |
| 43 | SOGA1 |  |  | 757 | -0.333 | 0.1231 | Yes |
| 44 | CPLANE1 |  |  | 762 | -0.335 | 0.1382 | Yes |
| 45 | CLTB |  |  | 816 | -0.349 | 0.1154 | No |
| 46 | STK39 |  |  | 858 | -0.359 | 0.1026 | No |
| 47 | FAM161A |  |  | 880 | -0.366 | 0.1059 | No |
| 48 | TBC1D5 |  |  | 958 | -0.398 | 0.0668 | No |
| 49 | TTYH3 |  |  | 1039 | -0.439 | 0.0275 | No |
| 50 | DLG1 |  |  | 1100 | -0.469 | 0.0056 | No |
| 51 | UPP1 |  |  | 1101 | -0.469 | 0.0312 | No |
| 52 | UBL3 |  |  | 1105 | -0.471 | 0.0545 | No |
| 53 | SETBP1 |  |  | 1106 | -0.471 | 0.0802 | No |
| 54 | HSPA4L |  |  | 1131 | -0.497 | 0.0884 | No |
| 55 | MDM2 |  |  | 1135 | -0.499 | 0.1132 | No |
| 56 | MYO6 |  |  | 1206 | -0.590 | 0.0901 | No |
Table: GSEA details [plain text format]

  

Fig 2: NB\_KI\_CLUSTER6: Random ES distribution      
 Gene set null distribution of ES for **NB\_KI\_CLUSTER6**
